# Circulating microRNA Expression Profiling Identifies miR-125a-5p Promoting T Helper 1 Cells Response in the Pathogenesis of Hashimoto's Thyroiditis

**DOI:** 10.3389/fimmu.2020.01195

**Published:** 2020-06-11

**Authors:** Yingzhao Liu, Xiangmei Ding, Si Xiong, Xuehua Wang, Xinyi Tang, Li Wang, Shengjun Wang, Huiyong Peng

**Affiliations:** ^1^Department of Endocrinology, The Affiliated People's Hospital of Jiangsu University, Zhenjiang Medical School of Nanjing Medical University, Zhenjiang, China; ^2^Department of Endocrinology, The Fifth People's Hospital of Wuhan, Wuhan, China; ^3^Division of Hematology and Internal Medicine, Mayo Clinic, Rochester, MN, United States; ^4^Department of Laboratory Medicine, The Affiliated People's Hospital of Jiangsu University, Zhenjiang Medical School of Nanjing Medical University, Zhenjiang, China

**Keywords:** hashimoto's thyroiditis, microRNA expression profiling, miR-125a-5p, T helper 1 cells, MAF, pathogenesis

## Abstract

MicroRNAs (miRNAs) have emerged as key regulators of cellular processes by suppressing target mRNAs at the posttranscriptional level. However, little is known regarding the expression of miRNAs in peripheral blood mononuclear cells (PBMCs) from Hashimoto's thyroiditis (HT) patients. Therefore, 38 HT patients and 36 healthy volunteers were enrolled in this study to identify HT-mediated changes in miRNA expression. Over 1,000 dysregulated miRNAs and their biological functions in the HT patients were identified. Among them, miR-125a-5p expression was upregulated and inversely correlated with low levels of MAF, a transcription factor that inhibits Th1 cells activity and the production of IFN-γ. Luciferase assay results demonstrated that MAF is a direct target gene of miR-125a-5p. Moreover, the proportion of circulating Th1 cells and the transcript levels of IFN-γ were increased in the HT patients. MiR-125a-5p expression positively correlated with the proportion of circulating Th1 cells and the serum concentrations of anti-thyroperoxidase antibodies in the HT patients. Interestingly, knockdown of miR-125a-5p in CD4^+^ T cells resulted in an elevated level of MAF but decreased the proportion of Th1 cells and the transcript level of IFN-γ *in vitro*. Furthermore, upregulated miR-125a-5p and IFN-γ transcript levels and downregulated MAF expression were detected in thyroid tissues from HT patients. Receiver operating characteristic (ROC) curves suggested that miR-125a-5p has a crucial role in the HT. Our results demonstrate that the elevated levels of miR-125a-5p contribute to the Th1 cells response in the HT patients and may be involved in the pathogenesis of HT.

## Introduction

Hashimoto's thyroiditis (HT), also known as chronic lymphocytic thyroiditis (CLT), is an organ-specific autoimmune disease that belongs to the spectrum of chronic autoimmune thyroid diseases (AITD) (1). HT patients typically exhibit infiltration of lymphocyte cells in the interstitium between thyroid follicles, a diffusely enlarged thyroid gland and elevation of autoantibody production, including anti-thyroglobulin antibody (TgAb) and anti-thyroperoxidase antibody (TPOAb) (2). Although patients can be euthyroid or even clinically manifest thyrotoxicosis, most of them ultimately develop hypothyroidism. HT was first described by Hakaru Hashimoto in 1912, and its prevalence exhibits an increasing trend (3). After more than a century, HT is now considered the most common autoimmune disease, endocrine disorder and cause of hypothyroidism (1). Although the results of studies suggest that cellular and humoral immune dysregulation are important pathogenic factors in the HT, the underlying pathogenesis remains poorly understood (4).

Non-coding RNAs are well-known to play crucial roles in the pathogenesis of HT (5, 6). MicroRNAs (miRNAs or miRs) are a class of small non-coding endogenous RNA molecules of ~22 nucleotides in length that are involved in the post-transcriptional regulation of gene expression. MiRNAs bind to the 3'-untranslated regions (3'-UTRs) of messenger RNAs (mRNAs) in a sequence-dependent manner and ultimately either repress translation or cause degradation of target mRNAs (7). MiRBase has an estimated 3,000 annotated human miRNAs that regulate approximately one-third of protein-coding genes (8). Accumulating evidence suggests that miRNAs play critical roles in the development of autoimmune diseases (9). Until recently, studies of the dysregulated expression of miRNAs in the HT have focused on plasma and thyroid tissue (10, 11). However, little is known regarding the expression profiles and functions of miRNAs in the peripheral blood mononuclear cells (PBMCs) from HT patients.

In this study, we aimed to identify miRNAs expression profiles in PBMCs from HT patients and the role of miRNAs in the pathogenesis of HT with next-generation high-throughput sequencing (NGS). Using this approach, we hoped to identify the potential roles of miRNAs in the HT patients.

## Materials and Methods

### Subjects and Samples

Twenty-three HT patients, 16 females and 7 males, were enrolled in this study. The primary clinical characteristics of these patients are shown in Table 1. All patients were diagnosed by clinical manifestation and auxiliary examination, including B-mode ultrasonography and laboratory criteria. The serum concentrations of free triiodothyronine (FT3) (3.28–6.47 pmol/L), free thyroxine (FT4) (7.64–16.03 pmol/L), thyroid stimulating hormone (TSH) (0.56–5.91 uIU/ml), TgAb (0–4 IU/ml) and TPOAb (0–9 IU/ml) were measured by chemiluminescent immunoassay using an LDX-800 instrument (BECKMAN COULTER, California, USA). Positive TgAb or TPOAb tests were obtained for all HT patients, nine of whom with hypothyroidism had a high serum TSH concentration, which in some cases was combined with a low level of serum FT4, while the remaining patients were euthyroid. Twenty-five age- and sex-matched healthy subjects were included as controls, all of whom were euthyroid and their thyroid-specific autoantibodies were within the normal range. They also had no history of thyroid diseases or other autoimmune diseases. The number of peripheral leukocytes in all individuals was within the normal range. For miRNA sequencing analysis, 5 patients and 5 healthy subjects were selected at random and 48 other samples (23 patients and 25 healthy subjects) were prepared for real-time fluorescent quantitative PCR (qRT-PCR) verification experiments. Peripheral blood samples were obtained from all patients and healthy subjects.

**Table 1:** Clinical features of HT patients and healthy subjects induced in the study.

	**HT patients**	**Healthy subjects**	**Range**
Number	23	25	
Gender (M/F)	7/16	8/17	
Age (year)	51 ± 15	49 ± 11	
FT3 (pmol/L)	4.86 ± 0.64	5.31 ± 0.61	3.28–6.47
FT4 (pmol/L)	10.72 ± 1.76	10.68 ± 1.28	7.64–16.03
TSH (uIU/ml)	6.34 ± 5.79	2.27 ± 0.97	0.56–5.91
TgAb (IU/ml)	95.8 ± 120.0	0.2 ± 0.5	0–4
TPOAb (IU/ml)	277.9 ± 346.0	1.3 ± 1.4	0–9

*Data correspond to the arithmetic mean ± SD and were compared using unpaired t-tests. M, male; F, female*.

Fresh tissue samples from the thyroid glands of 10 HT patients and 6 simple goiter patients were collected during thyroidectomy and stored at −80°C. Samples from simple goiter patients were used as control thyroid samples.

All patient sampling procedures were approved by the ethics committee of the Affiliated People's Hospital of Jiangsu University and collected at the Affiliated People's Hospital of Jiangsu University after informed consent was obtained from the subjects. All operations in this study adhered to standard biosecurity and institutional safety procedures.

### Cell Isolation and Purification

Human PBMCs were isolated by density-gradient centrifugation over Ficoll-Hypaque solution (Tianjin Haoyang Biological Technology Co., Tianjin, China) according to the manufacturer's instructions. Human CD4^+^ T cells were purified from PBMCs with magnetic beads using a CD4^+^ T cell Isolation Kit (Miltenyi Biotec GmbH, Bergisch Gladbach, Germany) according to the manufacturer's instructions. Human PBMCs and CD4^+^ T cells were cultured in RPMI-1640 medium (Gibco, California, USA) supplemented with 10% fetal bovine serum (Gibco). HEK293T cells were cultured with DMEM (Gibco) containing 10% fetal bovine serum (Gibco) at 37°C in 5% CO_2_. Thyroid specimens were minced and then digested with collagenase II (Sigma-Aldrich, California, USA) for 1–2 h at 37°C. Thyroid tissue cells were isolated by density-gradient centrifugation over Ficoll-Hypaque solution (Tianjin Haoyang Biological Technology Co.) according to the manufacturer's instructions.

### RNA Library Preparation and miRNA Sequencing

RNA library preparation and high-throughput sequencing were performed by Cloud-Seq Biotech Ltd. Co. (Cloud-Seq Biotech Ltd., Shanghai, China). A TruSeq Stranded Total RNA Library Prep lit (Illumina, USA) was used to pretreat RNA in preparation for the construction of the sequencing library. Library quality control and quantitative analysis were performed using a Bio-Analyzer 2100 system (Agilent Technologies, USA). The 10 pM libraries were denatured into single-stranded DNA molecules, captured on an Illumina Flowcell, amplified into clusters *in situ*, and sequenced for 150 cycles on an Illumina HiSeq 4000 sequencing instrument in two-terminal mode (PE mode).

### miRNA Profiling Analysis

Paired-end reads were obtained from the Illumina HiSeq 4000 sequencing instrument, and quality control was performed by assessing the Q30 quality score. High-quality reads were obtained by using CutAdapt (v1.9.3) to remove low-quality reads for the analysis of miRNAs and were subsequently compared with the human reference genome (UCSC HG19) using hisat2 software (v2.0.4). The FPKM (fragments per kilobase of exon per million fragments mapped) values of miRNAs, representing the miRNA expression profiles for the PBMCs of HT patients and healthy volunteers, were calculated using cuffdiff as described in the GTF gene annotation file. Moreover, the fold changes and *p*-values of statistical indicators between the experimental and control groups were calculated to identify miRNAs with significantly different expression levels (fold-change > 1.5 and *p* < 0.05). Gene Ontology (GO) and Kyoto Encyclopedia of Genes and Genomes (KEGG) analyses of differentially expressed miRNA-associated genes were performed to predict the function of miRNAs.

### RNA Extraction and qRT-PCR

Total RNA was isolated from PBMCs with TRIzol reagent (Invitrogen, California, USA) according to the manufacturer's instructions. RNA concentrations were measured using a NanoDrop ND-1000 instrument (Thermo Fisher Scientific, Waltham, MA, USA), while the quality of RNA was assessed by determining the OD260/280 ratio. RNA integrity was measured by modified agarose gel electrophoresis. Reverse transcription (Toyobo, Osaka, Japan) was performed to synthesize cDNA according to the manufacturer's instructions using the oligo-dT and random primers provided in the ReverTraAca® qPCR RT kit (Toyobo). qRT-PCR was performed in triplicate using TaKaRa TB Green™ Premix Ex Taq II (TaKaRa, Osaka, Japan). The primer sequences used for qRT-PCR were as follows: MAF, sense, 5′-TGGCAATGAGCAACTCCGAC-3′, antisense, 5′-CACTGGCTGATGATGCGGTC-3′; IFN-γ, sense, 5′-GAGTGTGGAGACCATCAAGGA-3′, antisense, 5′-TGTATTGCTTTGCGTTGGAC-3′; and β-actin, sense, 5′-CACGAAACTACCTTCAACTCC-3′, antisense, 5′-CATACTCCTGCTTGCTGATC-3′. MiRNA sequences were obtained from miRbase, and the primers used to amplify miRNAs were designed by RiboBio Co. (RiboBio, Guangzhou, China). The data were analyzed using Applied Biosystems 7500 Manager software.

### Flow Cytometry Analysis

Isolated PBMCs or CD4^+^ T cells were resuspended at 1 × 10^6^/ml in RPMI-1640 medium containing 10% fetal bovine serum and stimulated with 50 ng/ml phorbol myristate acetate (PMA; Sigma-Aldrich) and 1 μg/ml ionomycin (Sigma-Aldrich) for 2 h. Subsequently, the cells were incubated for an additional 4 h in the presence of 1 μg/ml brefeldin-A (eBioscience, San Diego, USA) at 37°C in 5% CO_2_. After incubation, the suspended cells were stained with relevant mAbs, including phycoerythrin-cyanin 5 (PE-Cy5)-conjugated anti-human CD3 mAb, fluorescein isothiocyanate (FITC)-conjugated anti-human CD8 mAb (eBioscience), PE-conjugated anti-human IFN-γ mAb, PE-conjugated anti-human IL-4 mAb (Miltenyi Biotec GmbH) and Alexa Fluor 488-conjugated anti-human c-MAF mAb (eBioscience). The data were analyzed using FlowJo 10 (Stanford University, San Francisco, USA). In this study, we defined CD3^+^ CD8^−^ IFN-γ^+^ cells as Th1 cells and CD3^+^ CD8^−^ IL-4^+^ cells as Th2 cells.

### miRNA Transfection

A miR-125a-5p-specific inhibitor and negative control (NC) (RiboBio) were designed to chemically modify target-specific miR-125a-5p in cells. The isolated human CD4^+^ T cells were transfected with miR-125a-5p or NC using Entranster-R (Engreen Biosystem, Co. Ltd., Beijing, China) according to the manufacturers' instructions for 48 h in the presence of 0.5 μg/ml functional anti-human CD3 Ab plus 2 μg/ml functional anti-human CD28 Ab (Miltenyi Biotec GmbH) before restimulation. Subsequently, the IFN-γ^+^ cells and IL-4^+^ cells were subjected to flow cytometric analysis.

### Luciferase Reporter Assay

The wild-type (WT) sequence of the MAF 3'UTR containing the miR-125a-5p binding site was generated by Sangon Biotech Co. (Sangon, Shanghai, China), as was a mutant unable to bind miR-125a-5p. The WT and mutated sequences of the MAF 3'UTR (NM_001031804) were cloned into the vector psiCHECK-2 (Promega, Madison, USA). Then, a dual-luciferase reporter plasmid and miR-125a-5p mimics, inhibitor or NC were cotransfected into HEK293T cells using Lipofectamine 3000 reagent (Thermo Fisher Scientific, Waltham, MA, USA) and Entranster-R in 24-well plates. Forty-eight hours after transfection, luciferase activity was measured using the Dual-Luciferase Reporter Assay System (Promega, Madison, USA) according to the manufacturers' instructions.

### Statistical Analysis

GraphPad Prism version 5 software (GraphPad Software, Inc., San Diego, USA) was used for the management and analysis of data. Student's unpaired *t*-test was performed for comparisons between two groups when variables pass the normal distribution test. Mann-Whitney U test was used to analyse the difference between the two groups of non-normal distribution data. Correlations between variables were determined by Pearson's correlation coefficient. A *p* < 0.05 was considered significant (^*^p < 0.05, ^**^p < 0.01, ^***^p < 0.001).

## Results

### miRNA Expression Profiles in HT Patients

**Figure 1:**
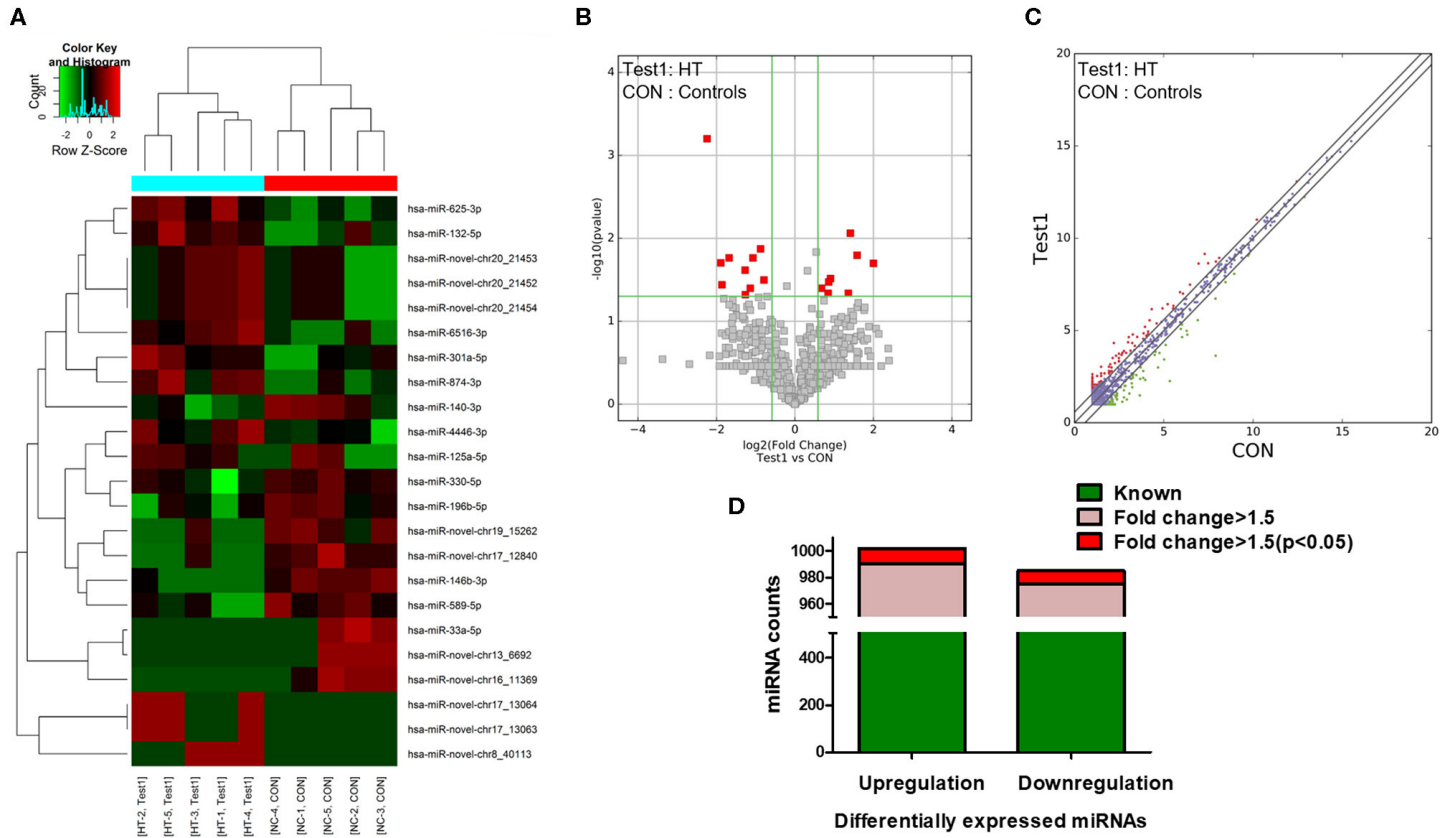
miRNA expression profiles in HT patients. **(A)** Hierarchical clustering analysis were performed to classified next generation sequencing (NGS) data of PBMCs and each square represented one patient or one healthy volunteer. Red and green represented upregulated and downregulated expression, respectively. HT indicates disease group; Con indicates control group. Volcano plots **(B)** and scatter plots **(C)** distinguished the expression patterns of different miRNAs. **(D)** Statistical analysis of differentially expressed miRNAs. Green represented differential expression, pink represented fold change > 1.5 of differential expression and red represented fold change > 1.5 (*p* < 0.05) of differential expression.

NGS was performed to identify human miRNAs (microarray GEO ID: GSE148157), and hierarchical clustering analysis results revealed different miRNA expression patterns in PBMCs from the HT and control samples (Figure 1A; red and green squares indicate up- and downregulated miRNAs, respectively). Volcano plots (Figure 1B) and scatter plots (Figure 1C) were generated to identify significantly and differentially expressed miRNAs. Using a 1.5-fold change as a cutoff, the results indicated that the levels of miRNAs in the HT samples were distinctly different from those in the control samples. A total of 1,310 dysregulated miRNAs were identified in PBMCs from HT patients and healthy volunteers, of which 659 were upregulated and 651 were downregulated. Among them, 655 miRNAs (331 upregulated and 324 downregulated) had a fold change > 1.5, and 22 miRNAs (12 upregulated and 10 downregulated) were verified as being significantly differentially expressed miRNAs (fold change > 1.5, *p* < 0.05) (Figure 1D). Although we identified many up- and downregulated miRNAs in the HT patients, only a small number of these differentially expressed miRNAs (12 upregulated and 10 downregulated) were significant from the sequencing data. One possible explanation for this result is that the sample size for NGS was too small. Therefore, we subsequently verified the sequencing results by expanding the sample size.

### Validation of Selected miRNAs by qRT-PCR

**Figure 2:**
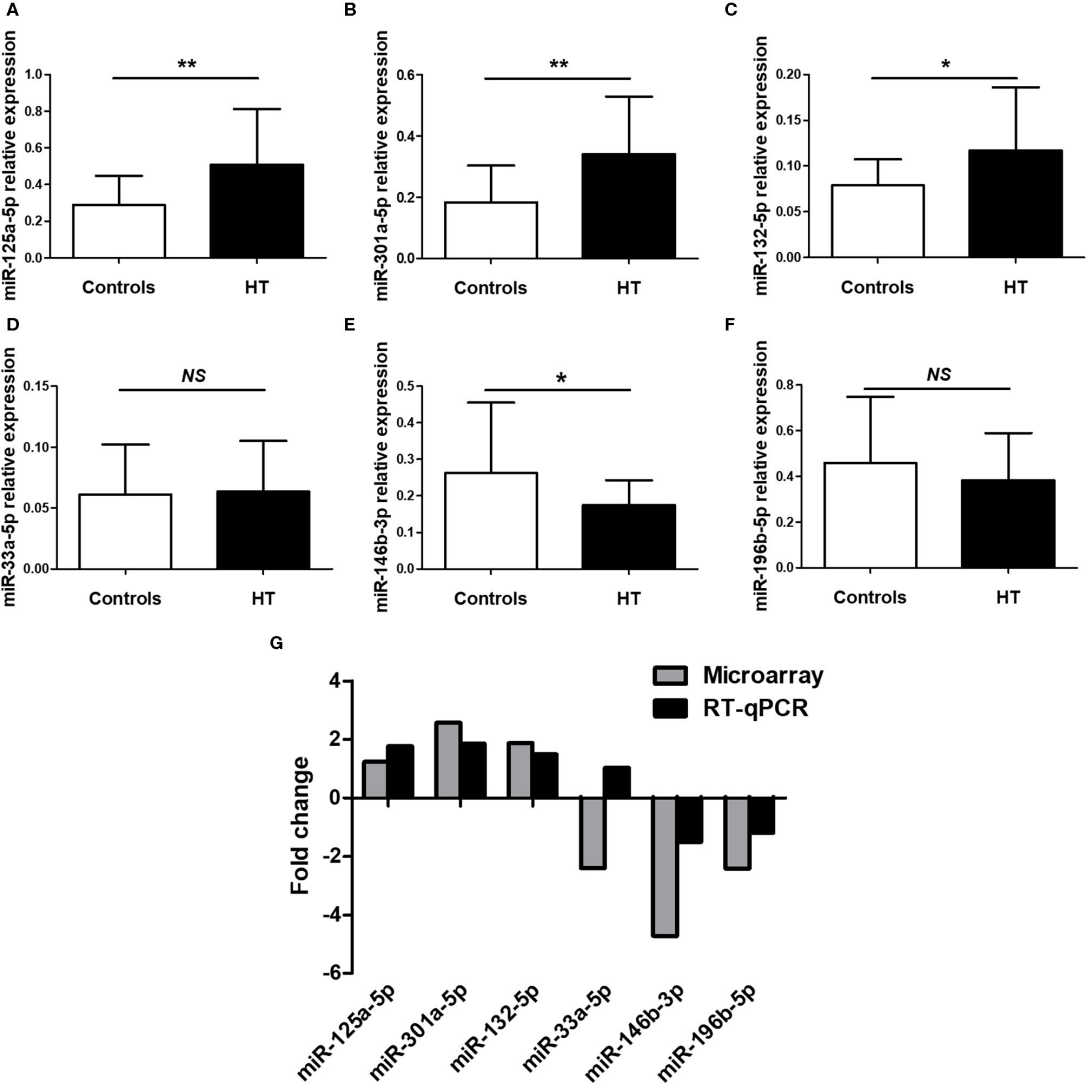
Validation of selected miRNAs by qRT-PCR. PBMCs was obtained from 23 HT patients and 25 healthy volunteers. The expression of miR-125a-5p **(A)**, miR-301a-5p **(B)**, miR-132-5p **(C)**, miR-33a-5p **(D)**, miR-146b-3p **(E)**, and miR-196b-5p **(F)** in the PBMCs from HT patients and healthy volunteers were determined by qRT-PCR. The fold changes of six selected miRNAs expression between qRT-PCR and NGS were determined **(G)**. Horizontal lines show the mean. *p < 0.05, **p < 0.01; NS, no significance.

To confirm the NGS data, we selected six differentially expressed miRNAs, 3 upregulated (miR-125a-5p, miR-301a-5p and miR-132-5p) and 3 downregulated (miR-33a-5p, miR-146b-3p and miR-196b-5p), based on the differential expression fold change, *p*-value and consistent dysregulated expression in each HT sample. qRT-PCR was performed to verify the expression of the selected miRNAs in PBMCs from 23 HT patients and 25 healthy controls. The results for miR-125a-5p, miR-301a-5p, miR-132-5p, miR-146b-3p, and miR-196b-5p were consistent with the NGS data, but only miR-125a-5p, miR-301a-5p, miR-132-5p, and miR-146b-3p expression achieved statistical significance. MiR-33a-5p expression was inconsistent with the trend observed in the NGS data (Figures 2A–F). The fold changes in the expression of the six selected miRNAs between qRT-PCR and NGS are shown in Figure 2G. The results indicated that the miRNA qRT-PCR and sequencing results were consistent at a rate of ~83%, but there were inconsistencies in the fold change and significance. Thus, it was necessary to verify the sequencing results by increasing the sample size.

### Predicted Functions and Pathways of Differentially Expressed miRNAs in HT

**Figure 3:**
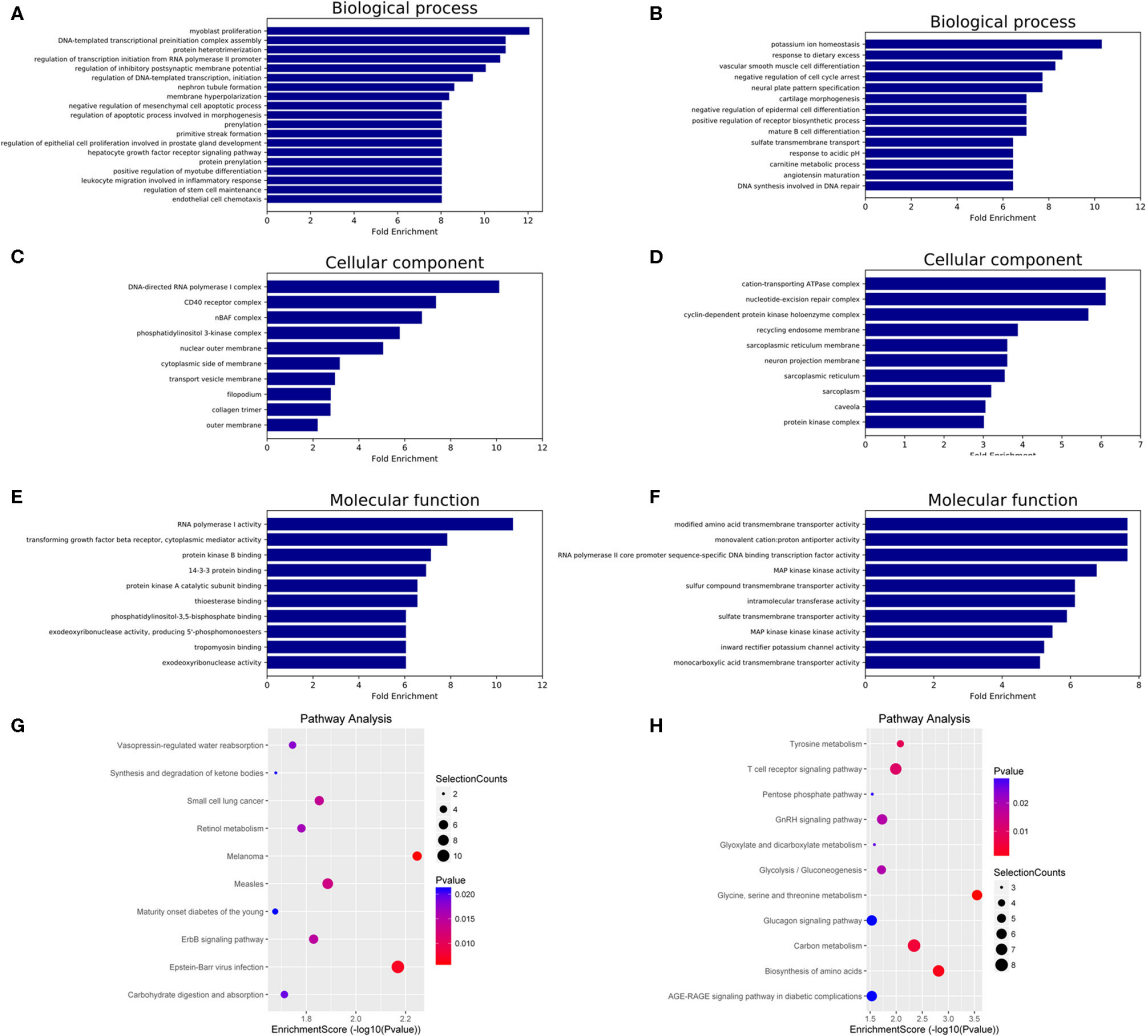
GO and KEGG pathway analysis of differentially expressed miRNAs in HT. GO enrichment analysis included biological processes, cellular components and molecular functions. The top 10 GO terms of source genes regulated by upregulated miRNAs **(A,C,E)** and downregulated miRNAs **(B,D,F)**. The top 10 KEGG pathways for upregulated miRNAs **(G)** and downregulated miRNAs **(H)**.

GO and KEGG analyses were performed to determine the potential biological functions of differentially expressed miRNAs. The GO enrichment analysis included biological process (BP), cellular component (CC) and molecular function (MF) categories. The top 10 GO enriched terms are shown in Figure 3. We observed that “myoblast proliferation,” “leukocyte migration involved in inflammatory response,” and “nephron tubule formation” were significantly regulated by overexpressed miRNAs in HT (Figure 3A). “CD40 receptor complex” and “phosphatidylinositol-3,5-bisphosphate binding” were also significantly regulated by these miRNAs (Figures 3C,E). Moreover, the GO term “MAP kinase kinase kinase activity” was associated with low miRNA expression (Figure 3F). These predicted GO terms regulated by miRNAs involve well-known functional genes associated with the pathogenesis of HT.

The KEGG analysis results identified 18 signaling pathways associated with potential target genes of upregulated miRNAs and 16 signaling pathways associated with downregulated miRNAs in HT, and the top 10 KEGG pathway terms of dysregulated miRNAs are shown in Figure 3G (upregulated miRNAs) and Figure 3H (downregulated miRNAs). Among these terms, “apoptosis,” “T cell receptor signaling pathway,” and “AGE-RAGE signaling pathway in diabetic complications” are involved in the regulation of the NF-κB, PI3K-Akt, MAPK, and Jak-STAT signaling pathways, which have been shown to participate in the pathogenesis of HT (Supplementary Figure 1, Supporting Information).

### Prediction of Potential miRNA Target Genes

**Figure 4:**
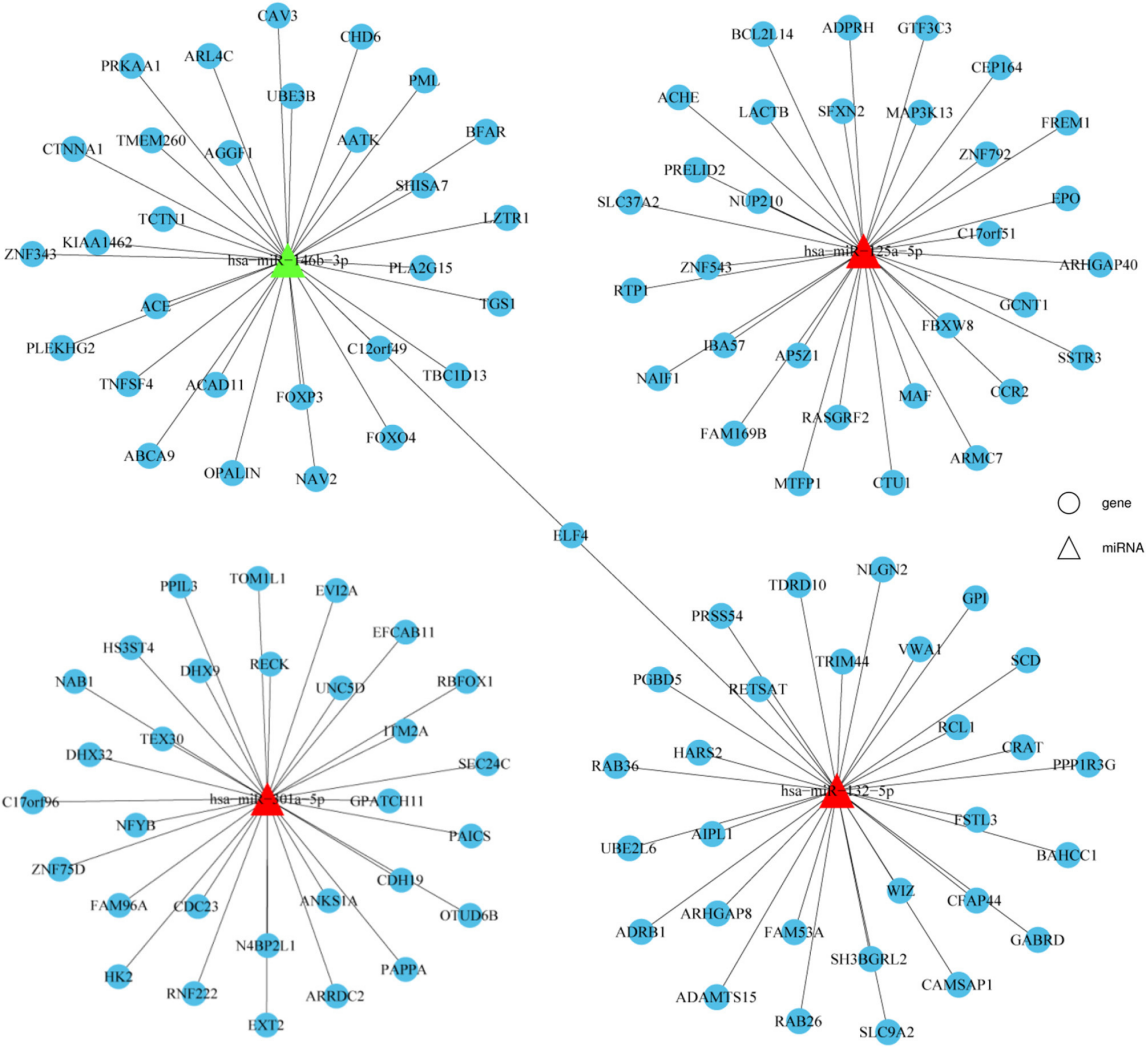
Prediction of potential miRNA target genes. Target genes prediction of verified miRNAs were performed by prediction software, including TargetScan and miRanda. The top 30 target genes were displayed by a distinct network diagram. Roundness indicated mRNA and Triangle indicated miRNAs.

Target gene prediction was performed to evaluate the potential functions of differentially expressed miRNAs in HT and to determine whether they can directly regulate gene expression. miR-125a-5p, miR-301a-5p, miR-132-5p, and miR-146b-3p were selected for this analysis due to the consistent results obtained between the NGS and qRT-PCR results. As shown in Figure 4, the top 30 potential target genes were selected according to the binding free energy and context^+^ scores between miRNAs and the 3'UTR binding sites of target genes. The information may aid in determining the mechanisms of the selected miRNAs.

### Correlation Between miR-125a-5p and MAF Expression in Peripheral Blood From HT Patients

**Figure 5:**
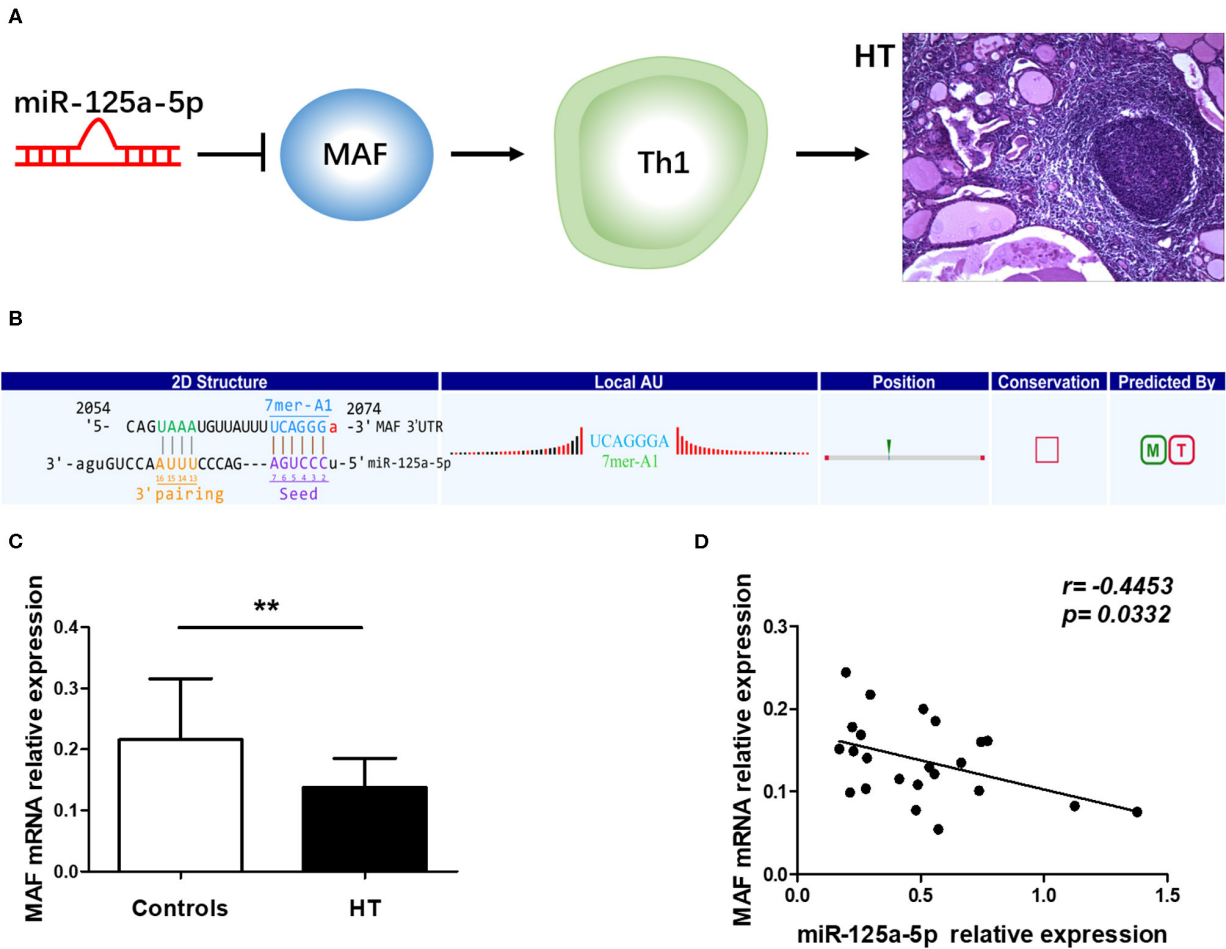
Correlation between miR-125a-5p and MAF in peripheral blood from HT patients. **(A)** Schematic diagram of potential mechanism of miR-125a-5p in HT. **(B)** The interactional sites between miR-125a-5p and the 3'UTR of MAF gene was displayed by prediction program. **(C)** The relative expression of MAF mRNA in the PBMCs from HT patients and healthy controls was determined by qRT-PCR. **(D)** The correlation between the levels of miR-125a-5p and the levels of MAF mRNA in the PBMCs from 23 HT patients. Each data point represents an individual subject, horizontal lines show the mean. **p < 0.01.

To determine the specific mechanisms associated with the verified miRNAs, we screened the predicted target genes that were potentially involved in the pathogenesis of HT. It was previously reported that MAF inhibits Th1 cell differentiation by suppressing the production of IFN-γ, and MAF has been shown to be downregulated in PBMCs from Multiple sclerosis (MS) patients (12, 13). Moreover, Th1 cells are highly associated with the pathogenesis of HT (14), and MAF was predicted to be a target of miR-125a-5p according to our sequencing results. Thus, we speculated that miR-125a-5p might promote the Th1 cells response by targeting MAF in HT (Figure 5A). To investigate the relationship between MAF and miR-125a-5p, MAF transcript levels were determined by qRT-PCR. As shown in Figure 5C, MAF transcript levels in PBMCs were decreased and inversely correlated with those of miR-125a-5p in HT patients (*r* = −0.4453; *p* = 0.0332) (Figure 5D). Moreover, one perfectly matched sequence of miR-125a-5p in the MAF 3'UTR was identified by prediction programmes (Figure 5B). These data suggested that the elevated levels of miR-125a-5p are associated with MAF expression in HT patients.

### MAF Is a Functional Target of miR-125a-5p

**Figure 6:**
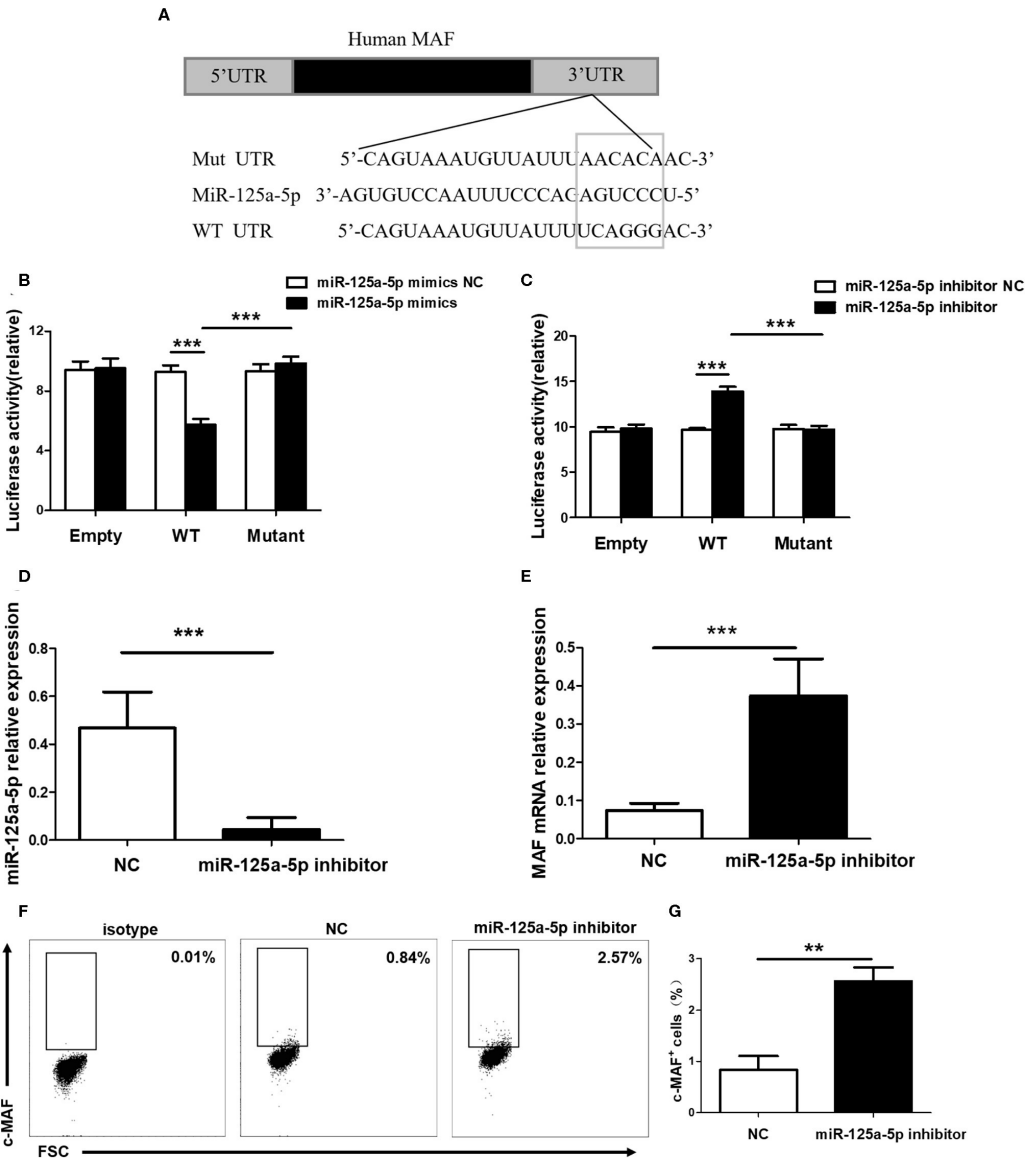
MAF is a functional target of miR-125a-5p. **(A)** MAF 3'UTR contained complementarities to miR-125a-5p seed regions using prediction programs analysis. **(B)** Luciferase activity of reporter carrying wild-type (WT) and mutant MAF 3'UTR and empty cotransfected into HEK293T cells with miR-125a-5p mimics and its negative control (NC). **(C)** Luciferase reporter activity in psiCHECK2 with WT and mutant MAF 3'UTR and empty cotransfected with miR-125a-5p inhibitor and its NC. MiR-125a-5p inhibitor and NC were transfected into human PBMCs, and the level of miR-125a-5p were detected by qRT-PCR **(D)**, and the level of MAF mRNA were detected by qRT-PCR **(E)**, and the percentage of c-MAF cells were analyzed by flow cytometry **(F,G)**. **p < 0.01; ***p < 0.001.

To determine whether miR-125a-5p directly targets MAF, WT and mutated MAF sequences were designed and synthesized (Figure 6A). Recombinant reporters were cotransfected into HEK293T cells with the miR-125a-5p mimics, the miR-125a-5p inhibitor or the NC. We found that the miR-125a-5p mimics inhibited the luciferase activity of the WT MAF 3'UTR reporter compared to that observed for the NC treatment, whereas the reporter with a mutated 3'UTR and unable to bind miR-125a-5p was unaffected by the miR-125a-5p mimics (Figure 6B). Moreover, the miR-125a-5p inhibitor enhanced the luciferase activity of the WT MAF 3'UTR reporter compared to that observed for the NC treatment but not for the reporter with the mutated 3'UTR (Figure 6C). Subsequently, to determine whether miR-125a-5p regulates MAF expression in human PBMCs, the miR-125a-5p inhibitor or NC was transfected into human PBMCs using Entranster-R. Treatment with the miR-125a-5p inhibitor resulted in a reduction in miR-125a-5p levels and an increase in MAF mRNA compared to that observed for the NC treatment (Figures 6D,E). Moreover, downregulated miR-125a-5p expression resulted in an increase in the percentage of c-MAF^+^ cells compared with that observed for the NC treatment (Figures 6F,G). Taken together, these data indicated that miR-125a-5p decreases MAF expression at both the transcriptional and translational levels.

### miR-125a-5p Contributes to Increased Circulating Th1 Cells in HT Patients

**Figure 7:**
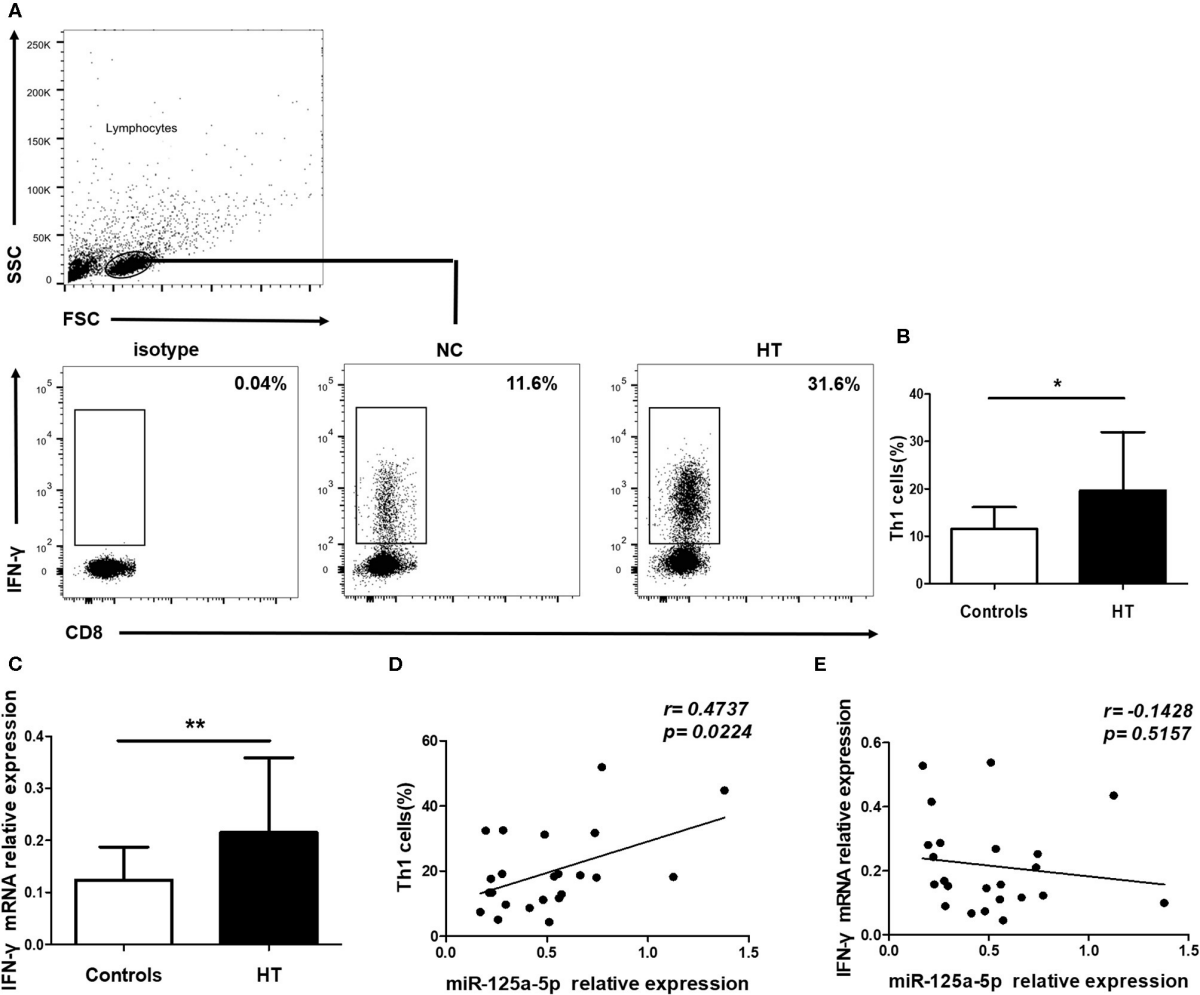
miR-125a-5p contributes to increased circulating Th1 cells in HT patients. Peripheral blood was obtained from 23 HT patients and 25 healthy volunteers. **(A)** Representative flow cytometry dot plots of Th1 cells were displayed. Values in the rectangular region corresponded to the percentage of Th1 cells. We used isotype control to determine the positive cells, and CD3^+^ CD8^−^ IFN-γ^+^ cells represented Th1 cells. **(B)** The proportion of Th1 cells were compared between HT patients and controls. **(C)** The relative expression of IFN- mRNA in the PBMCs from HT patients and healthy controls was determined by qRT-PCR. **(D)** The correlation between the levels of miR-125a-5p and the proportion of Th1 cells in peripheral blood from HT patients. **(E)** The correlation between the levels of miR-125a-5p and the transcript levels of IFN-γ mRNA in PBMCs from HT patients. Each data point represents an individual subject, horizontal lines show the mean. *p < 0.05; **p < 0.01.

To address the possibility that miR-125a-5p contributes to increased proportion of Th1 cells in HT patients, peripheral Th1 cells were first gated on CD3^+^ CD8^−^ cells as CD4^+^ T cells, owing to the downregulated expression of surface membrane CD4 molecules on human PBMCs after stimulation with PMA and ionomycin (15), and then IFN-γ^+^ cells were quantified by flow cytometric analysis (Figure 7A). The proportion of peripheral Th1 cells and the transcript levels of IFN-γ in PBMCs were increased in the HT patients (Figures 7B,C). Moreover, a positive correlation between the levels of miR-125a-5p and the proportion of Th1 cells was observed in the HT patients (*r* = 0.4737; *p* = 0.0224) (Figure 7D). However, no correlation was observed between the expression of miR-125a-5p and IFN-γ in PBMCs from HT patients (*r* = −0.1429; *p* = 0.5157) (Figure 7E). In contrast to the observed correlation between miR-125a-5p and the percentage of Th1 cells, no correlation was observed between the levels of miR-125a-5p and the increased proportion of CD8^+^ IFN-γ^+^ T cells (Supplementary Figures 2A,B, Supporting Information) from HT patients (Supplementary Figure 2C, Supporting Information).

**Figure 8:**
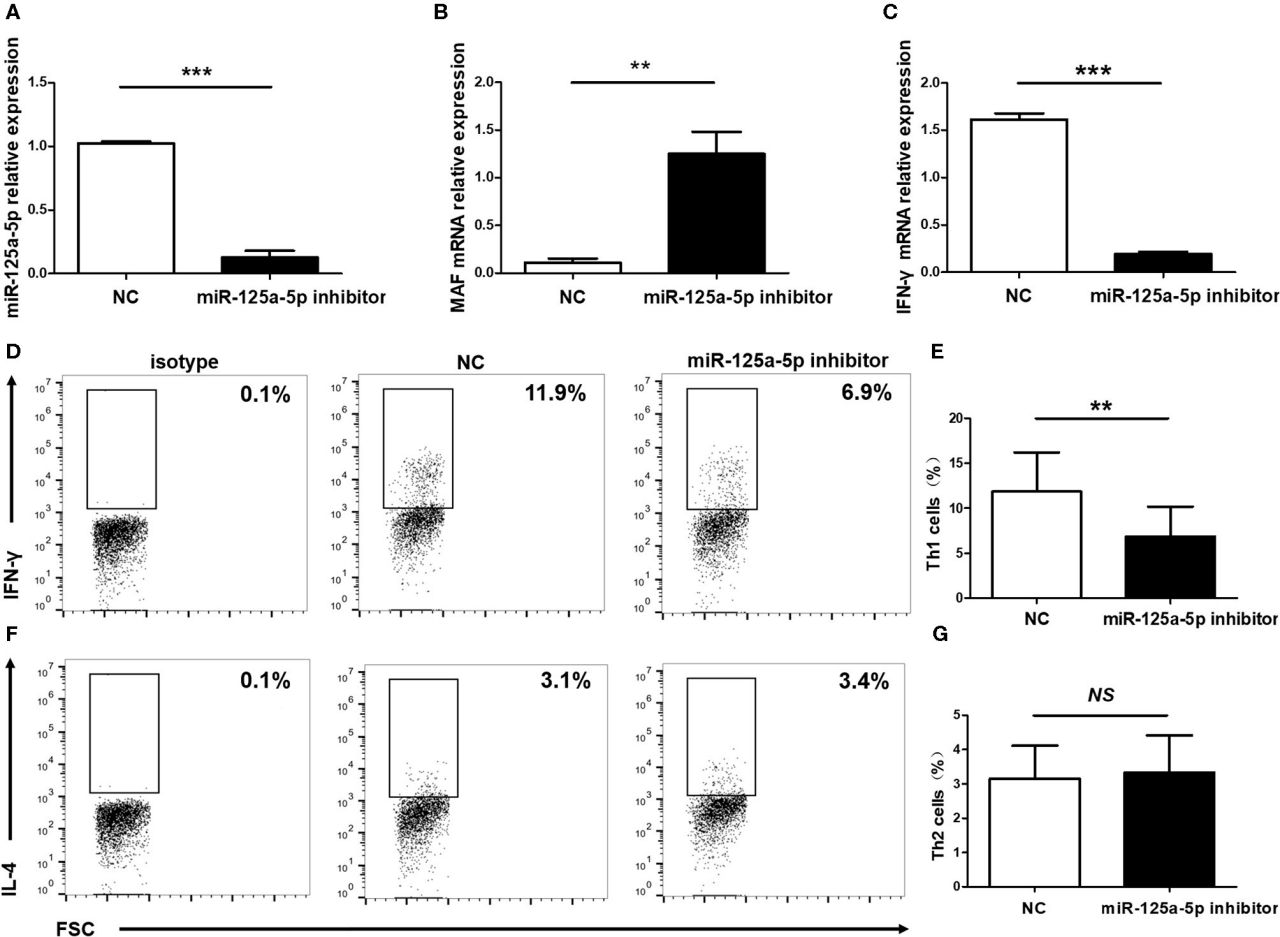
Influence of miR-125a-5p on Th1 cells *in vitro*. Human purified CD4^+^ T cells were isolated from PBMCs, and then transfected with miR-125a-5p inhibitor or NC. The transcript levels of miR-125a-5p **(A)**, MAF mRNA **(B)**, and IFN-γ mRNA **(C)** in CD4^+^ T cells between miR-125a-5p inhibitor and NC were detected by qRT-PCR. Downregulated expression of miR-125a-5p resulted in the reduction of the proportion of Th1 cells compared with that of the NC **(D,E)**. The proportion of Th2 cells between miR-125a-5p inhibitor and NC were analyzed by flow cytometry **(F,G)**. Each data point represents an individual subject, horizontal lines show the mean. **p < 0.01; ***p < 0.001; NS, no significance.

Subsequently, to determine the influence of miR-125a-5p on Th1 cells *in vitro*, purified human CD4^+^ T cells were transfected with the miR-125a-5p inhibitor or NC. The miR-125a-5p inhibitor downregulated the amount of miR-125a-5p and increased MAF transcript levels compared with that observed for the NC treatment (Figures 8A,B). Moreover, downregulation of miR-125a-5p decreased the proportion of Th1 cells and IFN-γ transcript levels in CD4^+^ T cells (Figures 8C–E). However, the proportion of Th2 cells was similar in the miR-125a-5p inhibitor and NC groups (Figures 8F,G). We also observed that downregulated miR-125a-5p expression was unable to reduce IFN-γ transcript levels in PBMCs (Supplementary Figure 3, Supporting Information). Taken together, these results indicate that miR-125a-5p promotes the Th1 cells response in the HT patients.

### MiR-125a-5p Expression in HT Patients Is Associated With Thyroid Antibodies

**Figure 9:**
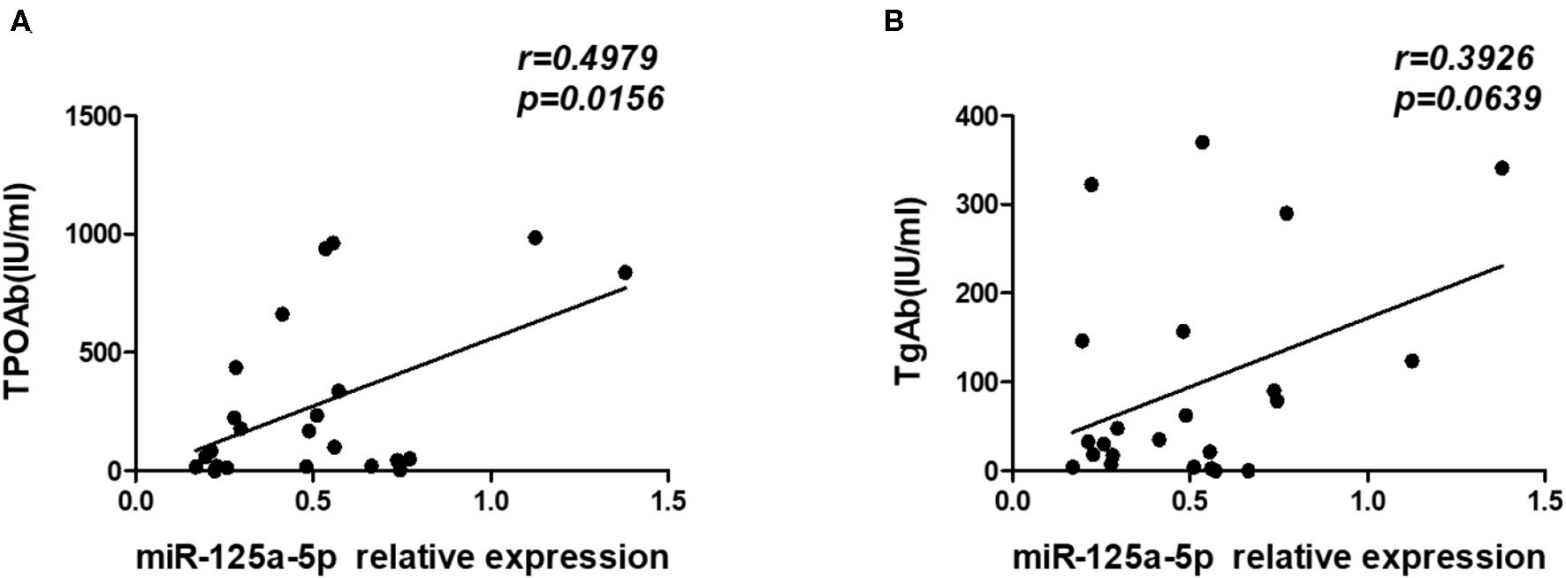
miR-125a-5p expression in HT associated with thyroid antibodies. The serum concentrations of TPOAb and TgAb from HT patients were detected by chemiluminescence. **(A)** The correlation between the levels of miR-125a-5p and serum concentrations of TPOAb in the HT patients. **(B)** The correlation between the levels of miR-125a-5p and serum concentrations of TgAb in the HT patients. Each data point represents an individual subject.

A distinguishing feature of HT is the presence of elevated levels of the thyroid antibodies TgAb and TPOAb. Therefore, we analyzed the relationship between miR-125a-5p and thyroid antibodies and observed that there was a significant positive correlation between miR-125a-5p and TPOAb (*r* = 0.4979; *p* = 0.0156) (Figure 9A). However, no correlation was observed between miR-125a-5p and TgAb (*r* = 0.3926; *p* = 0.0639) (Figure 9B). These data show that dysregulated miR-125a-5p expression is associated with the process of HT.

### MiR-125a-5p Expression in Thyroid Tissues From HT Patients

**Figure 10:**
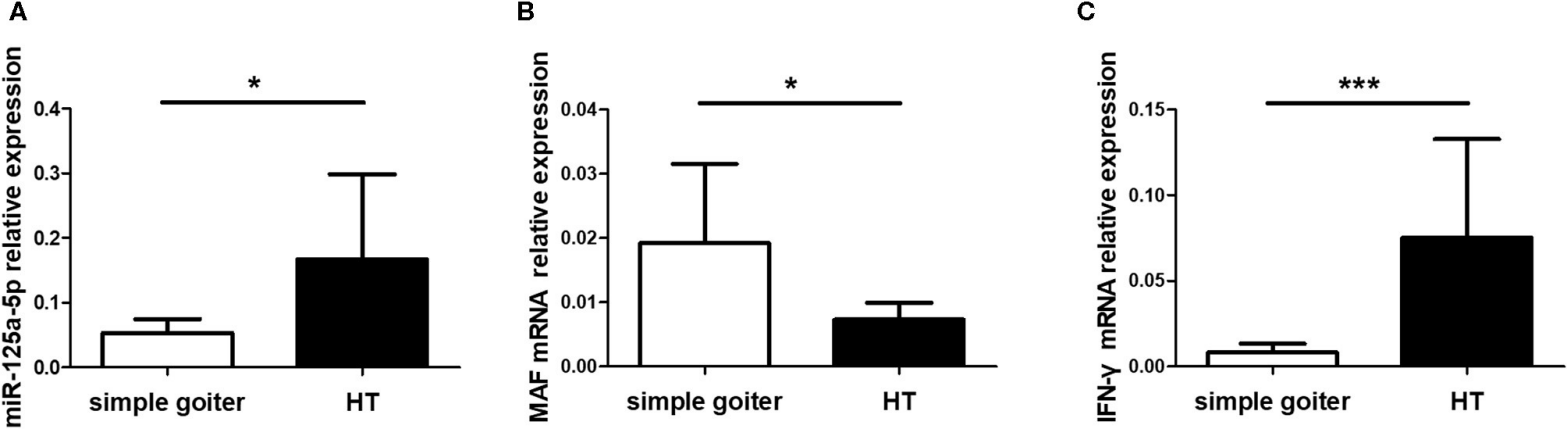
miR-125a-5p expressed in thyroid tissues from HT patients. Thyroid tissues were obtained from 10 HT patients and 6 simple goiter patients. The levels of miR-125a-5p **(A)**, MAF mRNA **(B)**, and IFN-γ mRNA **(C)** in thyroid tissues between HT patients and simple goiter patients were detected by qRT-PCR. Horizontal lines show the mean. *p < 0.05; ***p < 0.001.

As an organ-specific autoimmune disease, an understanding of the expression patterns between peripheral blood and thyroid tissues is crucial to functional and mechanistic research on HT. However, miR-125a-5p expression in thyroid tissues from HT patients remains unclear. Therefore, we assessed miR-125a-5p expression in thyroid tissues from HT patients and simple goiter patients. The elevated levels of miR-125a-5p (Figure 10A) and IFN-γ (Figure 10C) as well as decreased MAF expression (Figure 10B) were showed in HT patients.

### Potential Diagnostic Value of miR-125a-5p in HT Patients

**Figure 11:**
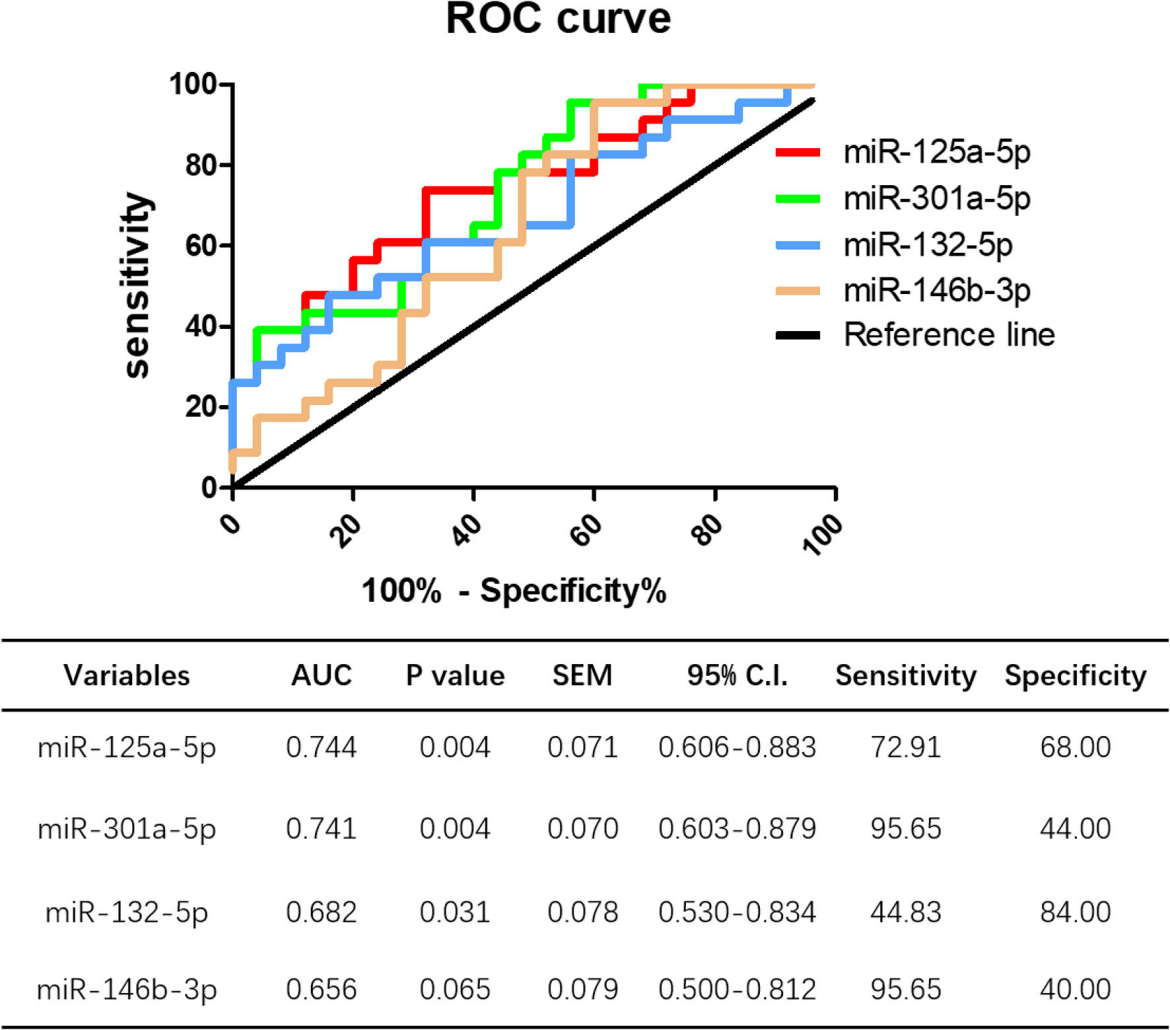
Potential diagnostic value of miR-125a-5p in HT. ROC curve analysis of miR-125a-5p, miR-301a-5p, miR-132-5p, and miR-146b-3p were performed to distinguish HT patients form healthy volunteer.

ROC curve analysis was performed to evaluate the potential diagnostic value miRNAs showing significantly different expression in peripheral blood between HT patients and healthy controls. The levels of miR-125a-5p, miR-301a-5p, and miR-132-5p could distinguish HT patients from healthy volunteers. Compared to miR-301a-5p (AUC 0.741, 95% CI = 0.603–0.879, *p* = 0.004), miR-132-5p (AUC 0.682, 95% CI = 0.530–0.834, *p* = 0.031), and miR-146b-3p (AUC 0.656, 95% CI = 0.500–0.812, *p* = 0.065), miR-125a-5p had the largest area under the ROC curve (AUC), up to 0.744 (95% CI = 0.606–0.833, *p* = 0.004), with a sensitivity and specificity of 72.91 and 68.00%, respectively (Figure 11). These data show that miR-125a-5p may be more valuable than the other identified miRNAs as a potential biomarker of HT.

## Discussion

The importance of aberrant miRNA expression is increasingly being recognized in autoimmune diseases, and the disease-associated miRNAs has become the key checkpoints of autoimmune diseases studies (16). In this study, we performed NGS to determine the miRNA profile of HT patients, the results of which identified 1,310 differentially expressed miRNAs (659 upregulated and 651 downregulated) in the PBMCs of HT patients. Among them, 22 miRNAs showed significantly dysregulated expression in HT patients. Some of these dysregulated miRNAs, including miR-132, miR-301a and miR-125a (overexpressed) (17–19) and miR-33a, miR-196b, and miR-146b (downregulated) (8, 20, 21), have been reported to be involved in immunological pathways or related to various autoimmune diseases. However, the relationships between these miRNAs and HT have rarely been reported. Immune dysregulation is known to be the primary cause of HT. Therefore, we speculated that these dysregulated miRNAs may be involved in the pathogenesis of HT by regulating the immune response.

Since only 5 samples used for NGS, which led to occasional inconsistencies, we subsequently verified miRNA candidates using an increased number of samples and observed that miR-125a-5p, miR-301a-5p, miR-132-5p, and miR-146b-3p were significantly differentially expressed in HT, which was consistent with the NGS data. To further explore the potential biological functions of these miRNAs, GO and KEGG enrichment analyses were performed to analyse the NGS data. Upregulated miRNAs were potentially associated with the GO terms myoblast proliferation (associated gene: HGF), CD40 receptor complex (associated gene: TRAF3), leukocyte migration involved in inflammatory response (associated gene: S100A9) and phosphatidylinositol-3,5-bisphosphate binding (associated gene: RAG2), which may participate in the occurrence of HT (22–25). In addition, Pax-8, which was associated with the GO term ‘nephron tubule formation’, participates in the pathogenetic process of AITD by regulating TG and TPO gene expression (26). SMARCA2, which is associated with vascular smooth muscle cell differentiation, is involved in apoptosis and thyroid volume in HT patients (27). Moreover, 34 signaling pathways associated with dysregulated miRNAs were identified by KEGG analysis. Among them, the NF-κB, PI3K-Akt, Jak-STAT, and MAPK signaling pathways were shown to be associated with the inflammatory response of HT (28–31). These data further indicate the accuracy of the NGS data and provide potential biological functions for miRNAs related to HT.

Generally, miRNAs are believed to function through complete or incomplete complementary binding with the 3'UTRs of target genes, and a network of miRNAs directly or indirectly controls the expression of target genes in a one-to-many or many-to-one manner (32). Our results identified 123 predicted target genes of miR-125a-5p, miR-301a-5p, miR-132-5p, and miR-146b-3p for further study. It has also become clear that miRNAs play multiple roles in the negative regulation of immune checkpoints (18, 33). Therefore, we wanted to determine whether the dysregulated miRNAs mentioned above are involved in the pathogenesis of HT by regulating specific immune checkpoints.

The Maf proto-oncogene, a basic region/leucine zipper transcription factor, is a pleiotropic regulator of T cell effector programming. As a member of the AP-1 transcription factor family, Maf is essential for the activation or repression of key immune checkpoints in CD4^+^ T cells, for the commitment of IL-17-producing γδ T cells (34–37), and for the differentiation and function of regulatory T cells in iTreg-Th17 homeostasis (38). Studies have shown that Maf, and its human ortholog, MAF, are downregulated in mouse and human Th1 cells, respectively, which impair Th1 differentiation and inhibit the production of IFN-γ (13, 39). A potential mechanism for the decreased Maf expression observed in Th1 cells is the low levels of histone H3 lysine 4 trimethylation (H3K4me3) and RNA polymerase II occupancy at the Maf promoter region (40). However, the mechanism has not been fully elucidated. Intriguingly, after extensive screening, MAF gene was identified as a potential target of miR-125a-5p. Since HT is a Th1-associated disease, we speculated that miR-125a-5p-mediated changes in MAF expression may be involved in the pathogenesis of HT. To confirm this hypothesis, firstly, prediction programmes were used to identify miR-125a-5p binding sites in the MAF 3'UTR, and an inverse correlation between increased levels of miR-125a-5p and decreased levels of MAF were detected in the PBMCs from HT patients. Secondly, miR-125a-5p was observed to inhibit the luciferase activity of a reporter containing the WT MAF 3'UTR but not that of a reporter with a mutated 3'UTR that is unable to bind miR-125a-5p, and the opposite phenomenon was observed in cells transfected with miR-125a-5p inhibitor. Finally, miR-125a-5p knockdown resulted in a considerable increase in MAF transcription and translation levels. These data suggest that miR-125a-5p directly regulates the expression of MAF in HT patients.

Subsequently, we wanted to determine whether miR-125a-5p can regulate Th1 cells. As expected, a significant positive correlation was observed between the levels of miR-125a-5p and the increased proportion of Th1 cells in HT patients. However, no correlation was observed between miR-125a-5p and IFN-γ transcript levels in the PBMCs from HT patients. One possible interpretation for this result is that activated CD8^+^ T cells, belonging to PBMCs, also secrete IFN-γ. Although our data showed that the miR-125a-5p inhibitor decreased the proportion of Th1 cells and IFN-γ transcript levels in purified CD4^+^ T cells, no correlation was observed between the levels of miR-125a-5p and the percentage of CD8^+^IFN-γ^+^ T cells, which was also increased in the PBMCs from HT patients. Moreover, PBMCs transfected with the miR-125a-5p inhibitor could not inhibit the expression of IFN-γ *in vitro*. Another possible interpretation is the involvement of different Maf activities between Th1 and CD8^+^ T cells. Maf has been shown to be induced in CD8^+^ T cells of type 2 NOD mice, which also have an increased propensity to produce IFN-γ. This activity may induce an overall type 1-biased immune response by inhibiting Maf-dependent DNA binding activities rather than the mode of action of Maf has in Th1 cells (41). Another study showed that Maf was expressed in CD8 cells and enhanced CD8 cells susceptibility to apoptosis by transactivating caspase 6 (42). However, the underlying mechanisms remain to be further elucidated.

Maf is highly expressed in both mouse and human Th2 cells, and its homodimers can bind to a Maf response element (MARE) in the proximal IL-4 promoter (12, 43, 44). Since miR-125a-5p was shown to directly regulate MAF expression, we hypothesized that miR-125a-5p knockdown resulted in an increased proportion of human Th2 cells. However, the percentage of Th2 cells was similar between cells transfected with the miR-125a-5p inhibitor and NC. One possible explanation for this result is that overexpression of MAF by the miR-125a-5p inhibitor attenuates the Th1 pathway through IL-4-independent mechanisms in human Th cells. Maf upregulation has been reported to skew naive CD4^+^ T cells toward the Th2 pathway to increase the production of IL-4 and decrease the production of IFN-γ (12, 35). However, we noted that miR-125a-5p transfection of CD4^+^ T cells also impaired the production of IFN-γ. In a previous study, the ectopic expression of Maf in mouse mature Th1 cells did not confer the ability to produce IL-4 but did attenuate IFN-γ production (13). The influence of Maf on Th1/Th2 polarization may be traced back to very early Th cell differentiation, even the Th0 stage. Another potential explanation is that MAF alone does not drive IL-4 expression but may need to synergize with other Th2-specific transcriptional factors, such as GATA-3 and NF-AT (45, 46). A similar phenomenon has been observed in Jurkat T cells, which produce endogenous IL-4 owing to the coexpression of MAF and GATA-3 (47, 48). Additional mechanisms that could explain the results of this study will be investigated in the future.

HT is an organ-specific autoimmune disease, and prior to this study, the expression of miR-125a-5p and MAF in the thyroid tissues of HT patients remained unclear. Compared with that detected in simple goiter patients, increased miR-125a-5p and IFN-γ expression and decreased MAF expression were observed in HT patients. Elevated serum concentrations of TgAb and TPOAb are widely accepted as the most common manifestations of HT in patients. Our data showed that miR-125a-5p expression was positively correlated with the serum levels of TPOAb but not TgAb. This result may be due to the different human IgG subclasses of TPOAb and TgAb. TPOAb and TgAb are represented by all four human IgG subclasses, while IFN-γ production by human Th1 cells only drives the generation of subclass IgG1 (49). These results suggest that miR-125a-5p expression, to some extent, indicates the disease severity of HT. In addition, we assessed the potential of miR-125a-5p as a diagnostic marker for HT, and the results indicated that miR-125a-5p could potentially differentiate HT patients from healthy controls, which combined with thyroid autoantibody levels could improve the diagnosis of HT.

The results of this study elucidated the expression profiles and potential biological functions of miRNAs in HT and demonstrated that miR-125a-5p regulates Th1 cells by directly targeting MAF. In HT patients, miR-125a-5p expression is significantly increased and correlates with the proportion of Th1 cells and disease severity. However, this study had some limitations. For instance, the relative expression of miR-125a-5p in PBMCs was obtained from a small number of patients and controls. Studies have shown that HT is more common in Whites and Asians than in African Americans (1). The associated study of miR-125a-5p and HT in Chinese Han Population, and our conclusions do not reflect all ethnic groups. The differences of miR-125a-5p expression among different regions, race and ethnicity deserve further study. In addition, we need to assess miR-125a-5p expression in hypothyroid patients and euthyroid patients. Taken together, our findings suggest that miR-125a-5p may contribute to the pathogenic role of the Th1 cells response in HT patients, although additional studies with larger cohorts of HT patients are needed.

## Data Availability Statement

The datasets generated for this study can be found in the GEO/GSE148157, https://www.ncbi.nlm.nih.gov/geo/query/acc.cgi?acc=GSE148157.

## Ethics Statement

The studies involving human participants were reviewed and approved by The Ethics Committee of the Affiliated People's Hospital of Jiangsu University. The patients/participants provided their written informed consent to participate in this study. Written informed consent was obtained from the individual(s) for the publication of any potentially identifiable images or data included in this article.

## Author Contributions

YL and XD carried out experiments, analyzed data, and wrote manuscript. SX, XW, and LW, helped with experiments and analyzed data. SW and XT participated in the design of experiments. HP planned experiments and supervised all the work on this paper. All authors discussed the results and commented on the manuscript.

## Conflict of Interest

The authors declare that the research was conducted in the absence of any commercial or financial relationships that could be construed as a potential conflict of interest.

## Supplementary Material

Supplementary Figure 1 KEGG analysis was performed to reveal the signaling pathways, which were involved in the pathogenesis of HT, including Apoptosis, T cell receptor signaling pathway and AGE-RAGE signaling pathway in diabetic complications. These signaling pathways were associated with of NF-κB, PI3K-Akt, MAPK, and Jak-STAT, which have been proved to play a crucial role in pathogenesis of HT.

Supplementary Figure 2 The correlation between the levels of miR-125a-5p and CD8^+^ IFN-γ^+^ T cells in HT patients. **(A)** Representative flow cytometry dot plots of CD8^+^ IFN-γ^+^ T cells in HT patients and healthy controls. Values in the upper right rectangular region corresponded to the proportion of CD8^+^ IFN-γ^+^ T cells. **(B)** The proportion of CD8^+^ IFN-γ^+^ T cells in PBMCs of HT patients and healthy volunteers were detected by flow cytometric analysis. **(C)** The correlation between the levels of miR-125a-5p and the proportion of CD8^+^ IFN-γ^+^ T cells in the HT patients. Each data point represents an individual subject, horizontal lines show the mean. ^**^*p* < 0.01.

Supplementary Figure 3 The transcript level of IFN-γ mRNA in PBMCs between miR-125a-5p inhibitor and NC were detected by qRT-PCR. Horizontal lines show the mean. NS, no significance.
