# Supplementary figures and images for: Circulating microRNA Expression Profiling Identifies miR-125a-5p Promoting T Helper 1 Cells Response in the Pathogenesis of Hashimoto's Thyroiditis
Source: Front Immunol. 2020 Jun 11;11:1195. doi: 10.3389/fimmu.2020.01195 (PMC7300231; doi:10.3389/fimmu.2020.01195)

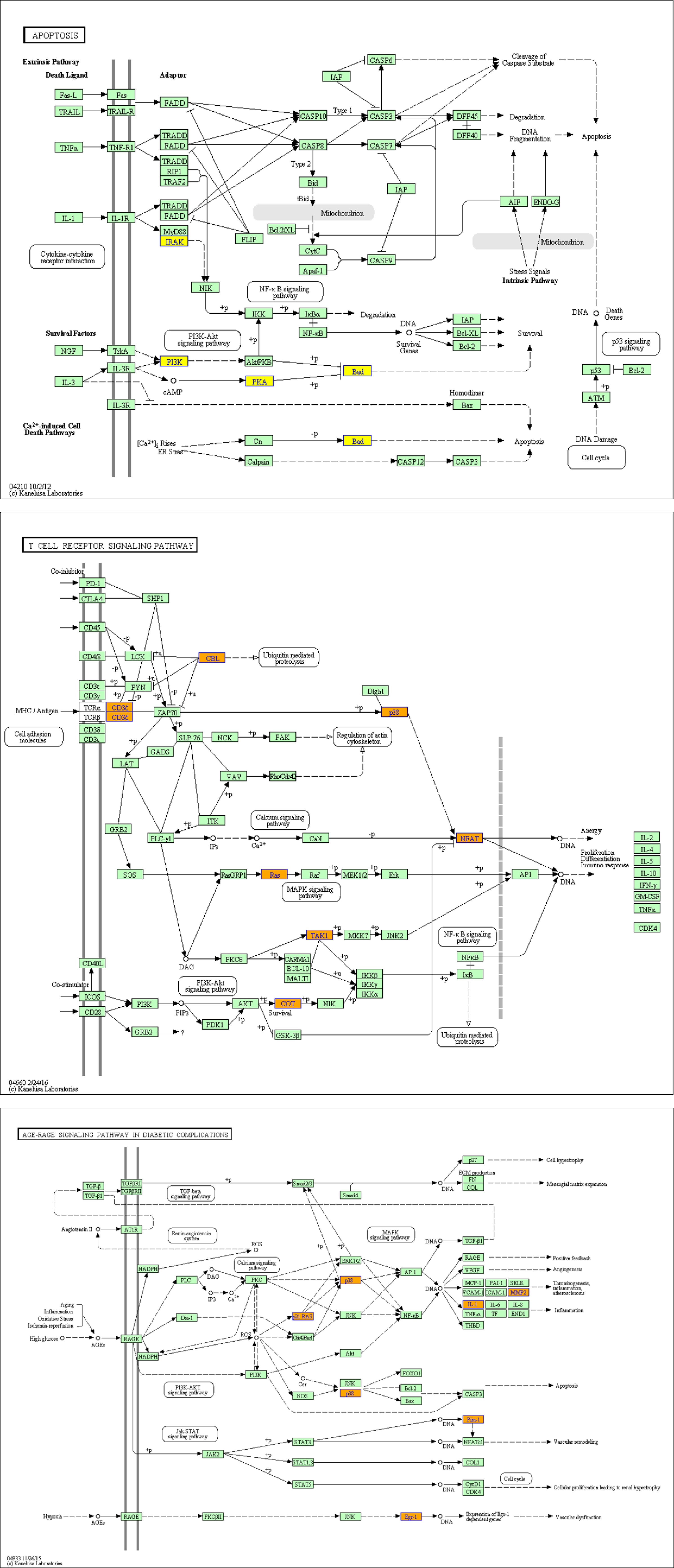

Supplement: Supplementary Figure 1 — KEGG analysis was performed to reveal the signaling pathways, which were involved in the pathogenesis of HT, including Apoptosis, T cell receptor signaling pathway and AGE-RAGE signaling pathway in diabetic complications. These signaling pathways were associated with of NF-κB, PI3K-Akt, MAPK, and Jak-STAT, which have been proved to play a crucial role in pathogenesis of HT. [file Image_1.TIFF]

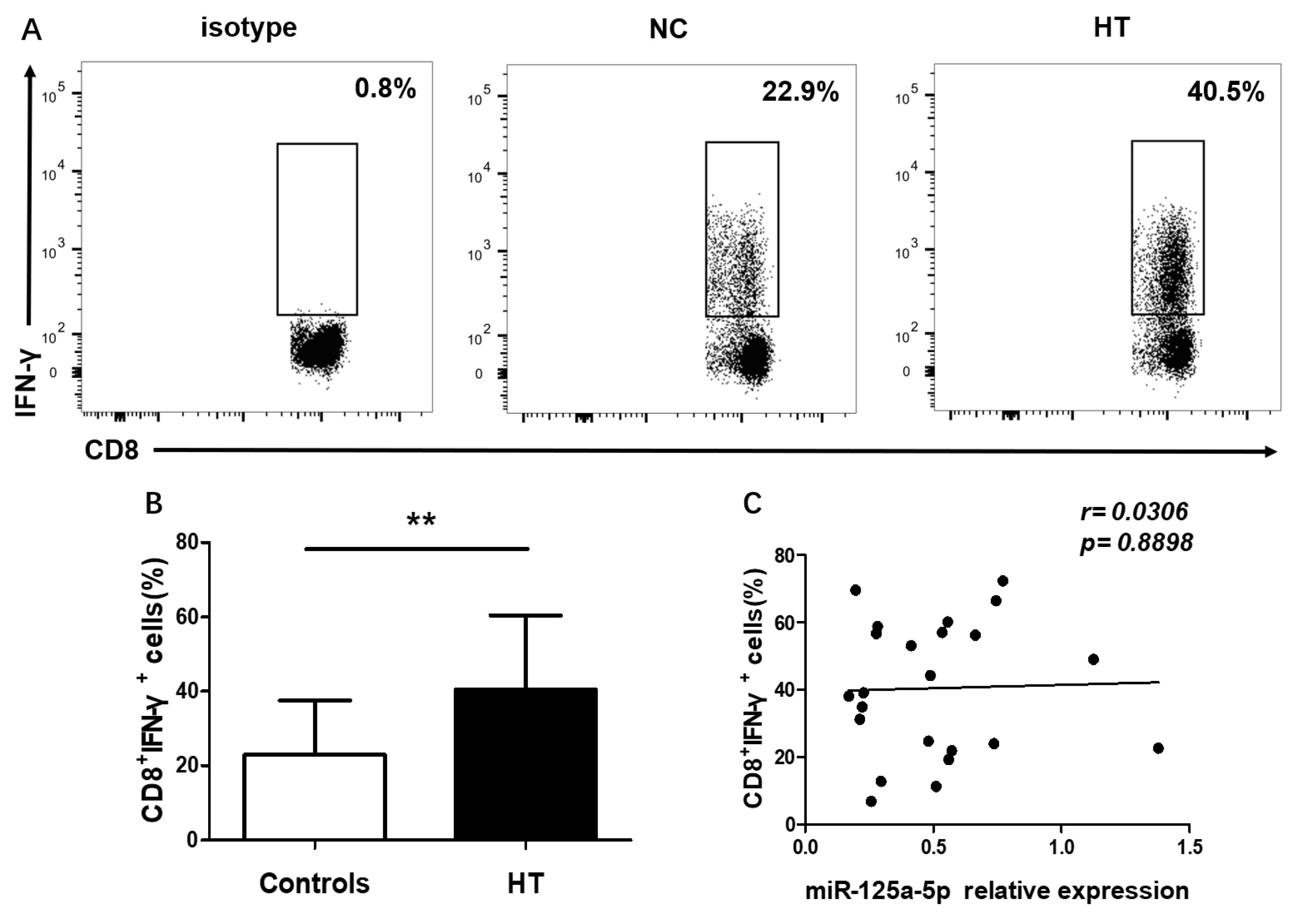

Supplement: Supplementary Figure 2 — The correlation between the levels of miR-125a-5p and CD8+ IFN-γ+ T cells in HT patients. (A) Representative flow cytometry dot plots of CD8+ IFN-γ+ T cells in HT patients and healthy controls. Values in the upper right rectangular region corresponded to the proportion of CD8+ IFN-γ+ T cells. (B) The proportion of CD8+ IFN-γ+ T cells in PBMCs of HT patients and healthy volunteers were detected by flow cytometric analysis. (C) The correlation between the levels of miR-125a-5p and the proportion of CD8+ IFN-γ+ T cells in the HT patients. Each data point represents an individual subject, horizontal lines show the mean. **p < 0.01. [file Image_2.TIFF]

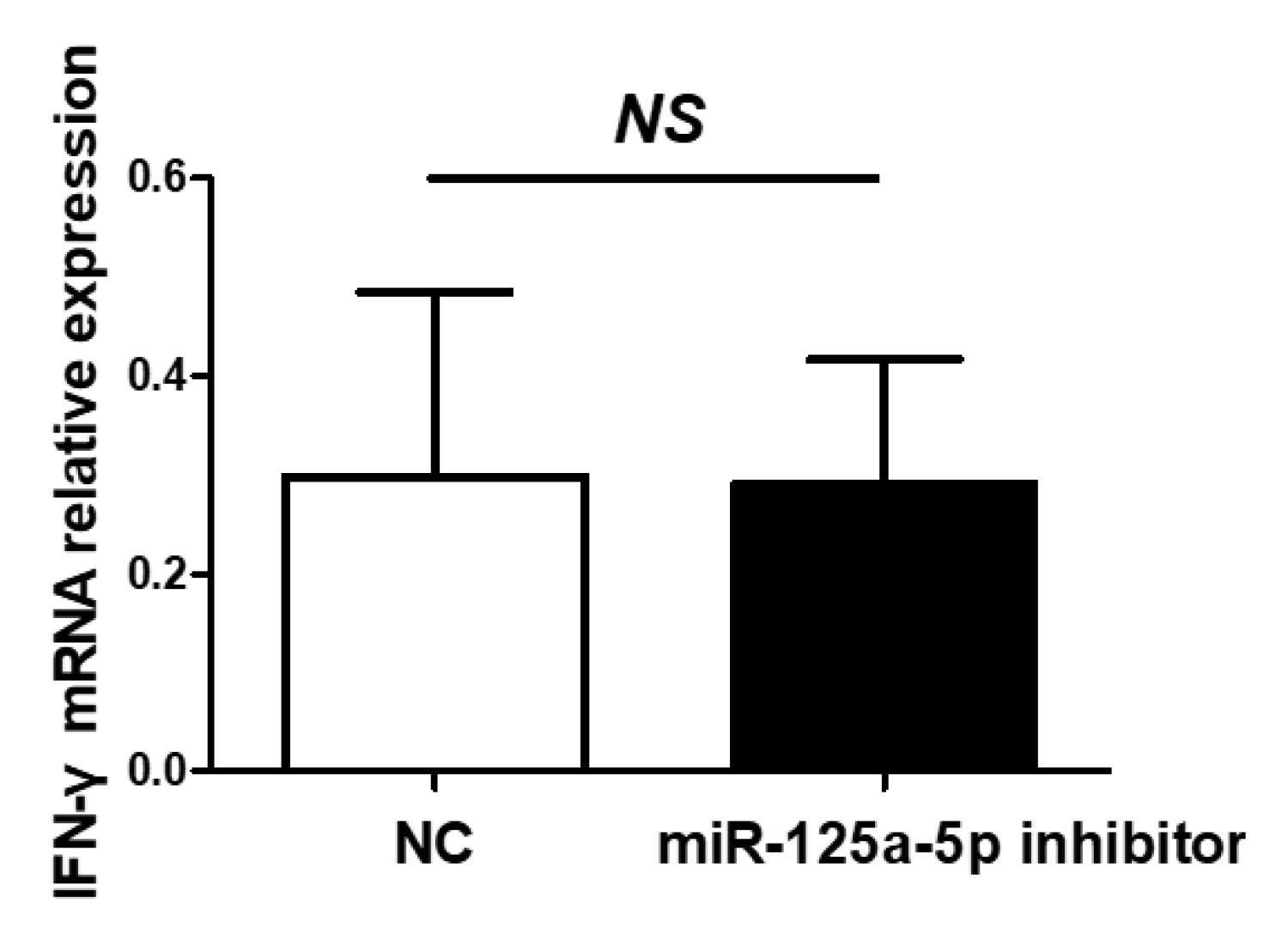

Supplement: Supplementary Figure 3 — The transcript level of IFN-γ mRNA in PBMCs between miR-125a-5p inhibitor and NC were detected by qRT-PCR. Horizontal lines show the mean. NS, no significance. [file Image_3.TIFF]
